# *In vivo* measurement of regional corneal tangent modulus

**DOI:** 10.1038/s41598-017-14750-w

**Published:** 2017-11-02

**Authors:** Ying Hon, Guo-Zhen Chen, Shu-Hao Lu, David CC Lam, Andrew KC Lam

**Affiliations:** 1 Laboratory of Experimental Optometry, School of Optometry, The Hong Kong Polytechnic University, Hung Hom, Hong Kong; 2 Department of Mechanical and Aerospace Engineering, The Hong Kong University of Science and Technology, Clear Water Bay, Hong Kong

## Abstract

Currently available clinical devices are unable to measure corneal biomechanics other than at the central region. Corneal stiffness (*S*), thickness, and radius of curvature was measured at the central cornea (primary fixation) and 3 mm from the temporal limbus (primary and nasal fixations). The corneal tangent modulus (*E*) of 25 healthy subjects was calculated from these data. After confirming normality, repeated measures analysis of variance (RMANOVA) revealed significant difference in *S* (*F*(2, 48) = 21.36, *p* < 0.001) at different corneal regions and direction of fixations. *E* also varied significantly at different corneal regions and direction of fixations (RMANOVA: *F*(2, 48) = 23.06, *p* < 0.001). A higher *S* and a lower *E* were observed at the temporal region compared with the corneal centre. Nasal fixation further increased *S* and *E* values compared with primary fixation. Due to the specific arrangement of corneal collagen fibrils, heterogeneity of corneal biomechanical properties is expected. In future clinical practice, localized corneal biomechanical alternation and measurement might assist corneal disease detection and post-surgery management. In addition, practitioners should be aware of the fixation effect on corneal biomechanical measurement.

## Introduction

The stroma is highly collagenous, comprising most of the corneal thickness, and thus, it mainly defines the biomechanical properties of the cornea. The stromal collagen fibrils exhibit preferential orientations. In the central region, lamellae of collagen fibrils run in either the superior-inferior or the nasal-temporal direction. These fibrils bend in the peripheral region and form the circumferential annulus^1–3^. In addition, the number of stromal lamellae increases from approximately 300 at the central cornea to 500 near the limbus^3^. Due to the different collagen orientation and density, regional variation in corneal biomechanical properties are expected and have been observed in human and bovine corneas in laboratory. Corneal biomechanical properties were measured as corneal elastic modulus, where a larger force is required to deform a material with higher elastic modulus. In an early report, Reichel *et al*.^4^ conducted strip extensiometry by cutting 2 × 7 mm corneal strips at the corneal centre and 3 mm from the corneal-scleral junction of bovine eyes. Higher elastic modulus was found in peripheral corneal tissue, supporting the presence of circumferential collagen banding in this region. Later studies adopting pressure inflation testing, found that corneal elastic modulus was higher in the central and paracentral corneal regions and lower in the peripheral and limbal regions^5,6^. The changes of elastic modulus were obviously different when measurements were taken meridionally and circumferentially. The highest meridional modulus was found in the central region, whilst the highest circumferential modulus was at the limbus^5,7^.

For most clinical applications, corneal biomechanical properties are measured using the Ocular Response Analyzer^8^ (ORA; Reichert Inc., USA) or the Corneal Visualization Scheimpflug Technology^9^ (Corvis ST; Oculus, Germany). They are modified pneumotonometers that record moments of applanation and quantify deformation responses only at the corneal centre. Most studies using the ORA reported an inability to distinguish keratoconus (KC) or suspected KC from normal eyes using its empirically-derived corneal biomechanical parameters, due to the wide overlap in their values^10–13^. The recently-developed Corvis ST provides corneal deformation parameters for analyzing corneal biomechanics. However, considerable overlap of values still exists^14–16^ and further studies have been performed to introduce corrected^17,18^ or customized parameters^19–21^ for increasing its sensitivity in disease detection. Nevertheless, clinical characteristics of keratoconus may not be limited to the central area^22^. Localized weakening of corneal biomechanical properties may be an important sign prior to the development of corneal ectasia^23^.

Recent clinical studies have demonstrated measurement of corneal tangent modulus *in vivo*, by applying corneal indentation at the central cornea^24–26^. The purpose of this study was to determine corneal tangent modulus at different corneal regions. Corneal indentation was applied at the central cornea and 3 mm from the temporal limbus. The effect of direction of fixation during eccentric corneal biomechanical measurement was also explored.

## Results

Of the 25 subjects enrolled in the study, the measurement results from both eyes are presented in Table 1.

**Table 1:** Between-eye comparison of ocular parameters for 25 subjects. The results are presented as mean ± standard deviation. CCT = central corneal thickness; TCT = temporal corneal thickness; *K* *c* = central corneal radius of curvature; *K* *t* = temporal corneal radius of curvature; CS = central corneal stiffness; TS = temporal corneal stiffness.

Parameter	Right eye	Left eye	Paired t-test	
CCT (µm)	536.7 ± 36.7	538.9 ± 35.6	*t* = −1.44,	*p = *0.16
TCT (µm)	625.6 ± 36.3	620.4 ± 36.4	*t* = 2.06,	*p* = 0.05
*K*_*c*_ (mm)	7.86 ± 0.25	7.86 ± 0.24	*t* = −0.20,	*p* = 0.85
*K*_*t*_ (mm)	8.41 ± 0.26	8.45 ± 0.24	*t* = −1.15,	*p* = 0.26
CS in primary fixation (N/mm)	0.070 ± 0.0065	0.068 ± 0.0068	*t* = 1.72,	*p* = 0.10
TS in primary fixation (N/mm)	0.074 ± 0.0074	0.072 ± 0.0087	*t* = 1.26,	*p* = 0.22
TS in nasal fixation (N/mm)	0.080 ± 0.0067	0.079 ± 0.0110	*t* = 0.44,	*p* = 0.67

**Figure 1:**
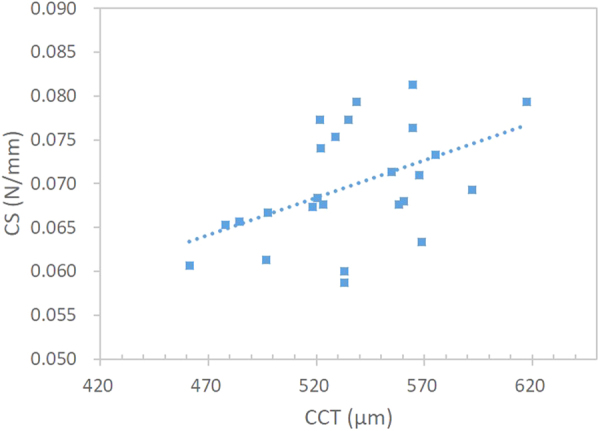
Central corneal stiffness (CS) was positively associated with central corneal thickness (CCT) (*r* = 0.48, *p* = 0.015).

**Figure 2:**
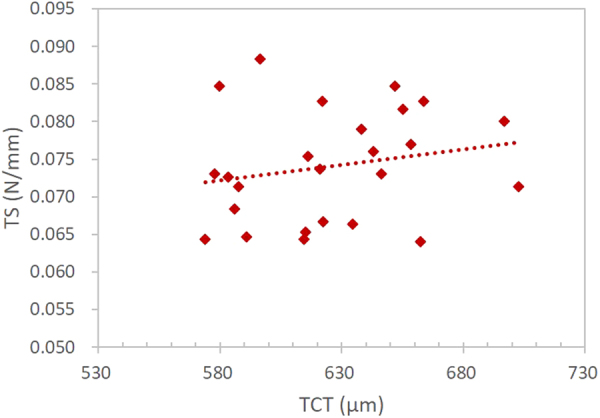
Temporal corneal stiffness (TS) was not significantly associated with temporal corneal thickness (TCT) (*r* = 0.20, *p* = 0.33).

**Figure 3:**
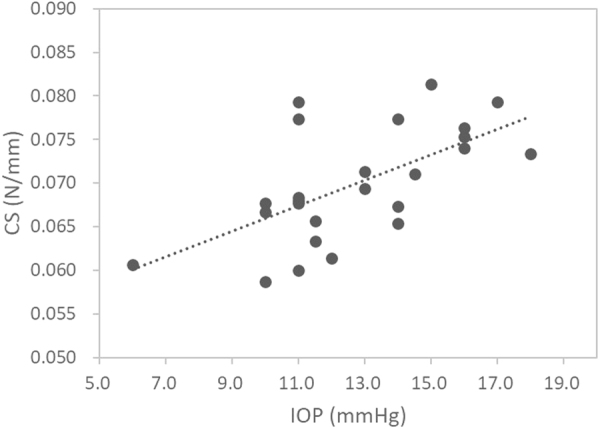
Central corneal stiffness (CS) was positively associated with intraocular pressure (IOP) (*r* = 0.62, *p* = 0.001).

Because there was no significant between-eye difference in CCT, TCT, *K*_*c*_, *K*_*t*_, CS, and TS, only the right eye results were used for further analysis. Corneal thickness and radius were significantly different between the central and peripheral regions. The human cornea was found to be thicker (*t* = −29.05, *p* < 0.001) and flatter (*t* = −13.01, *p* < 0.001) towards the temporal periphery. Significant difference was observed in corneal stiffness measurements (*F*(2, 48) = 21.36, *p* < 0.001). Post hoc test revealed significant difference between CS and TS in primary fixation (*p* = 0.024), in which higher stiffness was exhibited at the periphery. Corneal stiffness had no significant association with corneal radius at neither the central (*r* = −0.12, *p* = 0.56) nor temporal regions (*r* = 0.087, *p = *0.68). On the other hand, CS was positively associated with CCT (*r* = 0.48, *p* = 0.015) (Fig. 1), but correlation between TS and TCT was not significant (*r* = 0.20, *p* = 0.33) (Fig. 2). Since CS also demonstrated significant association with intraocular pressure (IOP) (*r* = 0.62, *p* = 0.001) (Fig. 3), multivariate analysis involving CCT and IOP showed that CS was dependent on IOP (partial *r* = 0.49, *p* = 0.015), but not on CCT (partial *r* = 0.25, *p* = 0.25).

Post hoc tests revealed significant difference between TS in different directions of fixation (p = 0.007). Nasal fixation yielded higher stiffness readings at the periphery compared with primary fixation.

The mean central *E* was 0.57 ± 0.07 MPa. The mean temporal *E* was 0.49 ± 0.07 MPa and 0.53 ± 0.06 MPa in primary and nasal fixation, respectively. Significant difference was revealed in corneal *E* measurements (*F*(2, 48) = 23.06, *p* < 0.001). Corneal *E* showed significant regional difference under primary fixation (*p* < 0.001). An average reduction of 13.7 ± 10.0% in corneal *E* was recorded at temporal periphery. In temporal measurements, corneal *E* also differed significantly in different directions of fixation (*p* = 0.008). Nasal fixation resulted in increased temporal *E* compared with primary fixation.

## Discussion

The current study attempted to measure corneal stiffness and tangent modulus at the central cornea and 3 mm from the temporal limbus using a novel corneal indentation device. The cornea is stiffer in the peripheral region than the central region. It is known that corneal overall stiffness can be influenced by corneal geometric parameters, such as thickness and curvature^27^, as well as the IOP^28,29^. Using the CID, it was determined that corneal stiffness increased with its thickness in the central cornea only. The insignificant association between temporal corneal stiffness and temporal corneal thickness could be due to limited sample size and data pool with normal young subjects. Nevertheless, statistical finding revealed that corneal stiffness was predominately influenced by IOP, rather than CCT. Thereby, the effect of IOP on corneal biomechanical measurement should be carefully considered in further studies.

At physiological IOP, a reduction of corneal tangent modulus by an average of 13.7 ± 10.0% was observed in the temporal quadrant of the peripheral cornea. In a recent study, distribution of the corneal tangent modulus was derived by monitoring topographic changes from temporary IOP elevation in human subjects^30^. With respect to the central measurement, these authors reported a mean reduction of 47.3% in corneal tangent modulus at a 2 mm wide peripheral annulus (4–6 mm away from the corneal apex). A small increase in corneal tangent modulus was also found in the paracentral cornea (2–3.5 mm away from the corneal apex). Due to the inherent difference in the applied load, direct comparison of measurement results could be difficult. But interestingly, both *in vivo* studies found reduced corneal tangent modulus around the peripheral cornea. The site of corneal indentation in our study was relatively closer to the corneal apex as compared with the selected peripheral annulus in Elsheikh *et al*.’s study^30^. The reduction in corneal tangent modulus could be less prominent.

Interestingly, it was found that peripheral corneal stiffness and tangent modulus measurement were influenced by direction of fixation. The extraocular muscles create an external force on the eye globe in different directions of gaze. During adduction, contraction of the medial rectus is accompanied by relaxation of the lateral rectus. However, tonic contraction of the lateral rectus and its stretching force at the muscle insertion, which is located about 6.9 mm from the corneal limbus^31^, could stress the scleral tissue resulting in an increased corneal stiffness and tangent modulus at the peripheral cornea. Another possible cause of rise in tangent modulus could be an increase in IOP during lateral gaze. Cooper *et al*.^32^. used an applanating transensor for continual monitoring of IOP under different conditions. Although the transensor measured resonant frequency rather than IOP directly, the variation of resonant frequency during extreme lateral gaze was equivalent to an IOP rise of 10 mmHg. Saunders *et al*.^33^. used a pneumotonometer to measure IOP of normal young adults under maximal peripheral fixations. IOP was found to be increased by 4.5 mmHg at extreme abduction. Nardi et al.^34^. found minimal changes in applanation IOP at lateral gaze. However, peripheral fixation was limited to 22° abduction. Moses *et al*.^35^. found an increase of 2 mmHg in Goldmann IOP during a 50-degree nasal fixation, which was similar to the current experimental protocol. Our study confirmed a positive association between corneal stiffness and IOP (Fig. 3). Although IOP was not measured during nasal fixation, the increase in corneal stiffness and tangent modulus due to IOP rise at adduction could not be ruled out. Hence, when attempting to measure corneal biomechanics using the CID in other corneal regions, practitioners should be aware of the effect from direction of fixation.

In view of the limitation of ORA and Corvis ST, the CID may be helpful in characterizing eccentric manifestations of keratoconus. In patients who had undergone myopic laser *in situ* keratomileusis with corneal collagen crosslinking, peripheral rebound tonometry was found comparable to the preoperative IOP taken at the central cornea^36^. With increasing popularity of corneal refractive surgeries, conventional tonometry for glaucoma screening and management is less accurate because of the measurement error induced by an alteration of corneal properties in the central region^10,37,38^. Corneal biomechanics outside the treatment zone might be preserved in the surgical process and hence peripheral tonometry might give a more predictable preoperative IOP. In short, the authors are encouraged by the potential application of regional corneal biomechanical assessment.

Regional corneal biomechanical measurement was limited to the temporal corneal region and involved a small sample size. The miniature size of the device could improve measurement flexibility and a large scale study is warranted to establish the regional variation of corneal tangent modulus in the human cornea. When conducting corneal biomechanical measurement away from the corneal centre, the influence due to scleral biomechanics should be considered. According to the assumption of corneal indentation method^39^, deformation due to indentation of a partial spherical shell by a concentrated force is not effected when a fixed boundary is about 2 mm or more from the site of indentation. As the limbus and sclera are flexible boundaries and corneal indentation was performed at 3 mm from the limbus, we attempted to reduce the scleral influence on corneal stiffness measurement.

To conclude, the current study demonstrated the feasibility of the CID to measure corneal stiffness and tangent modulus at central and temporal corneas. An increased corneal stiffness and reduced corneal tangent modulus were observed from the corneal centre to its periphery. Existing techniques for measuring regional elastic modulus of the cornea are either destructive or involve complex modeling. Corneal indentation is a technique that can be applied in a clinical setting. Practitioners should be aware of the effect from the direction of fixation during peripheral corneal biomechanical measurement.

## Methods

### Subjects

Twenty-five Chinese adults (14 men and 11 women) with an age range of 21 to 26 and good general health were enrolled. Exclusion criteria included inter-ocular difference in spherical equivalent refractive error **≥**3.00D, Goldmann applanation tonometry ≥21 mmHg, rigid lens wear, pregnancy, history of refractive surgery or eye disease, and use of long-term eye or oral medications. Soft lens wearers were required to cease contact lens wear for at least 24 hours before data collection. All procedures followed the Declaration of Helsinki and the protocol was reviewed and approved by the ethics review board of The Hong Kong Polytechnic University. Informed consent was obtained from each subject before commencement of the study. Data from both eyes was collected at a single visit. Noncontact procedures were conducted before contact procedures.

### Data collection

Corneal thickness and the corneal cross-sectional image were measured using a “3D Corneal Map” scan and a “2D Anterior Segment” scan respectively by swept-source anterior segment optical coherence tomography (AS-OCT; Casia SS-1000, Tomey Corp., Japan). Three automated measurements were obtained for each scan type, while the subject focused on a central target inside the instrument. Corneal radius of curvature was measured by corneal topography (E300, Medmont International Pty Ltd., Australia). Three images with scores higher than 95 were selected while the subject looked into the centre of the ring pattern inside the instrument.

**Figure 4:**
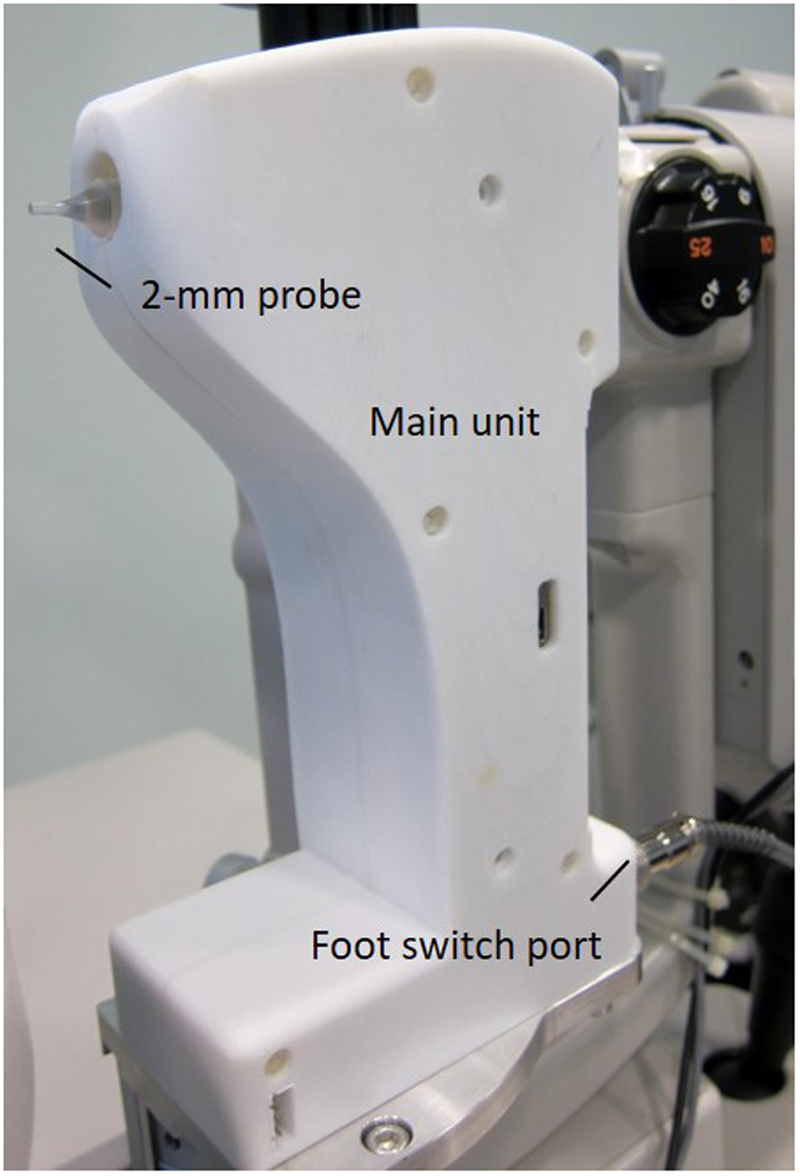
A photograph of the corneal indentation device (CID) mounted on a slit-lamp unit. It consists of a main unit, a 2-mm flat-faced indentation probe and a foot-switch.

**Figure 5:**
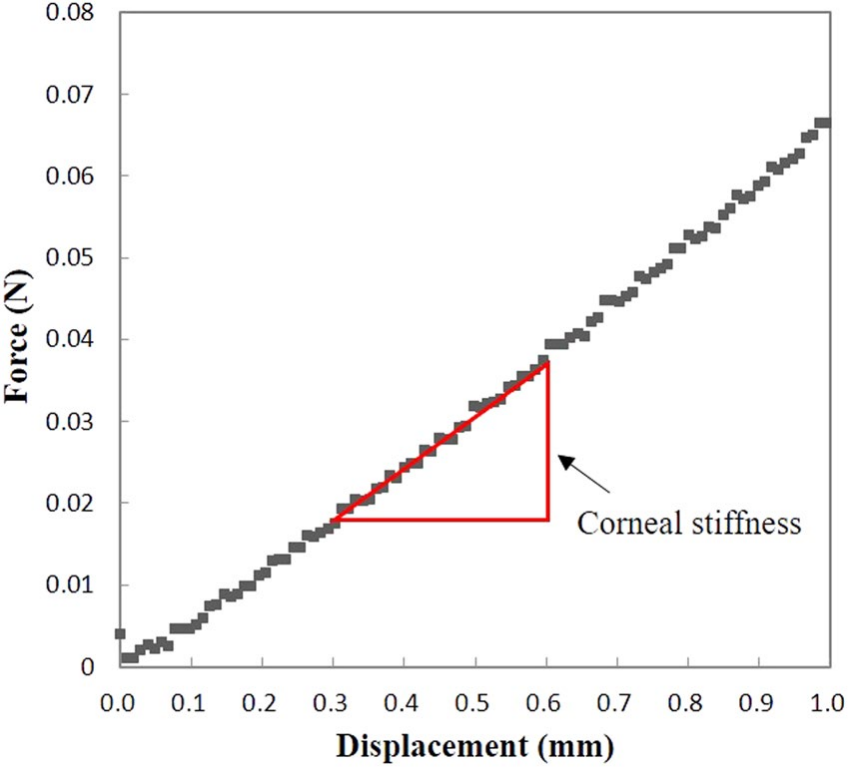
A real force-displacement curve from the corneal indentation device (CID). Corneal stiffness is the rate of change of force under a corneal displacement between 0.3–0.6 mm.

Corneal indentation was performed using a novel device (CID). Its methodology has been described in earlier work (details in Supplementary information)^40^. The current prototype was built to work with a slit-lamp biomicroscope (Fig. 4). It consists of a main unit, a 2-mm flat-faced indentation probe, and a foot-switch. Prior to the measurement, the probe was disinfected with 70% isopropyl alcohol, allowed to air-dry for 1 minute, and rinsed with normal saline. Following topical anaesthesia (one drop of 0.4% Benoxinate), the subject was instructed to rest his head and chin against the head and chin rests. The CID was reset and moved towards the cornea using a joystick from the slit-lamp. When the probe was in full contact to the cornea, a steady and low-pitched sound was issued indicating the readiness of data acquisition. By pressing the foot-switch, the probe was actuated forward at 12 mm/s to indent the cornea to 1 mm depth^41^. After reaching the set depth, the probe was immediately retracted from the cornea at the same rate. The entire measurement was completed in around 0.2 sec. The force required for corneal indentation was recorded and a force-displacement curve is shown in Fig. 5. A unique corneal biomechanical parameter obtained by the CID, corneal stiffness, was defined as the average rate of change of force under a corneal displacement between 0.3–0.6 mm.

**Figure 6:**
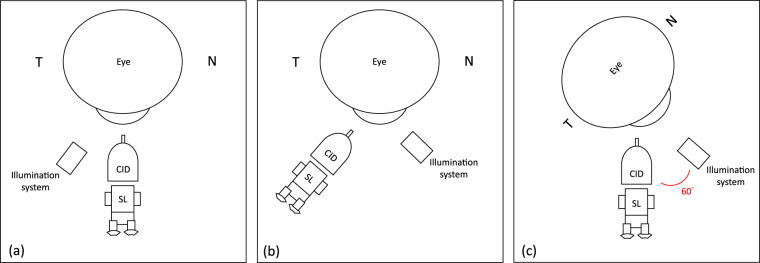
(**a**) A schematic diagram illustrates corneal stiffness measurement at central cornea; (**b**) at temporal cornea in primary fixation; (**c**) at temporal cornea in nasal fixation. T = temporal; N = nasal; CID = corneal indentation device; SL = slit-lamp.

Corneal indentation was applied randomly at the central cornea and 3 mm from the temporal limbus. Central corneal stiffness (CS) measurement was carried out at the corneal geometric centre while the subject looked straight ahead at an external target (CS in primary fixation) (Fig. 6a). Temporal corneal stiffness (TS) measurement was performed randomly using two fixation methods. In the first method, the CID was placed at the temporal side of subject’s eye while the subject looked straight ahead (TS in primary fixation) (Fig. 6b). In the second method, the CID was placed in front of the subject, while the illumination system of the slit-lamp was set at 60 degrees to the nasal side of his/her eye. The subject was instructed to look nasally and fixate on a target on the illumination system (TS in nasal fixation) (Fig. 6c). The location of measurement was the same in both fixation methods, whereby the indentation probe was placed 1-probe size away from the temporal limbus. Three valid readings were taken for each method.

As corneal biomechanics is pressure dependent^42^, IOP was measured at the central cornea by Goldmann applanation tonometer following corneal stiffness measurement.

**Figure 7:**
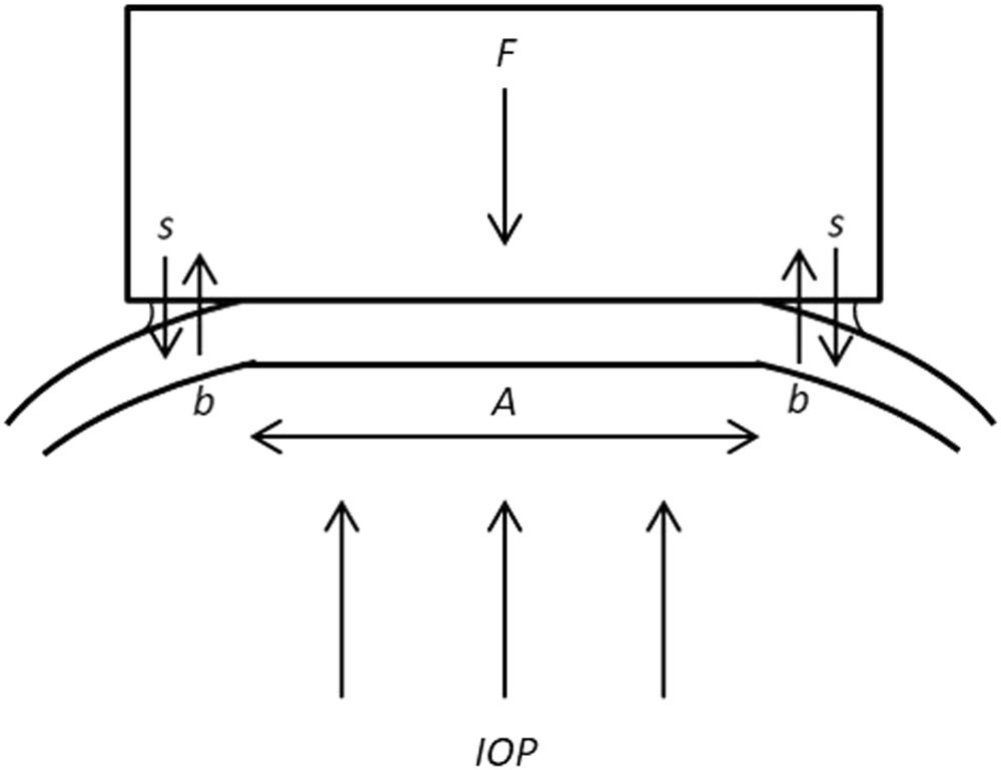
A force diagram representing corneal indentation, in which *F* is the applied force, *A* is the contact area of the cornea, *IOP* is the intraocular pressure, *s* is the surface tension of tear film and *b* is the resistance of the cornea to deformation.

### Treatment of data

#### Measurements of central and temporal corneal thickness and radius of curvature

Corneal thickness and radius at the site of indentation was retrieved from images captured by AS-OCT and Medmont topography.

Central corneal thickness (CCT) was readily shown in “3D Corneal Map” from AS-OCT. In order to obtain temporal corneal thickness (TCT), the temporal limbus was initially located in the En-face image from “2D Anterior Segment” scan and then relocated in the horizontal cross-sectional image from “2D Analysis” using the scale bar. From the corneal cross-sectional image, a 3-mm chord was drawn from the formerly located limbus into the cornea, in which the end point was indicated as the site of corneal indentation. The shortest horizontal distance *d* between the temporal limbus and the site of indentation was measured. Referring to the pachymetry map in “3D Corneal Map”, TCT could be obtained at a length of *d* from the visible temporal limbus into the cornea using the ruler tool.

Central corneal radius (*K*_*c*_) was calculated from averaging the simulated flattest and steepest keratometric readings shown in the tangential topographic image. Temporal corneal radius (*K*_*t*_) could also be obtained at a length of *d* from the visible temporal limbus into the cornea using the ruler tool.

#### Calculation of central and temporal corneal tangent modulus

According to equation (7) (in Supplementary information), corneal tangent modulus *E* at subject-specific IOP can be calculated by substituting the corresponding corneal stiffness, thickness, and radius measured at the site of indentation. Therefore, Central *E* was determined from CS, CCT, and *K*_*c*_ and Temporal *E* from TS, TCT, and *K*_*t*_. Temporal *E* in primary and nasal fixations were also calculated separately.

### Statistical analysis

Statistical analyses and graphics were performed using commercial software (SPSS 23.0, IBM Corp., USA & Microsoft PowerPoint 2016, Microsoft Corp., USA, respectively). All consecutive measurements were averaged for data analysis. The level of significance chosen was 5%. Shapiro-Wilk tests showed that all measured parameters were not significantly different from Gaussian distributions (*p* > 0.05). Hence, parametric tests were used to analyze the data.

Paired sample t-tests were performed to compare the between-eye differences in all measured parameters and also the difference in corneal geometry between the central and peripheral region. Repeated measures analyses of variance (RMANOVAs) were used to compare corneal stiffness measured in each method. Whenever significant differences were found, post-hoc comparisons were conducted with Bonferroni adjustment. Corneal biomechanical measurements could be confounded by corneal geometric parameters such as thickness and radius^27^. Bivariate correlation analyses were conducted to assess the relationships between corneal stiffness and corneal geometric parameters (thickness and radius) at central and temporal regions, respectively. Similar correlation analysis was performed to assess the relationship between central corneal stiffness and IOP at the central region. Subsequently, multiple linear regression was employed to further explore the association between central corneal stiffness with variables demonstrating significant correlations in the bivariate correlation analyses.

RMANOVAs were also used to compare corneal tangent modulus *E* measured in each method. Significant pairs were reported following Bonferroni adjustment.

## Electronic supplementary material

Mathematical deviations of corneal tangent modulus
