# Supplementary material for: In vivo measurement of regional corneal tangent modulus
Source: Sci Rep. 2017 Nov 2;7:14974. doi: 10.1038/s41598-017-14750-w (PMC5668273; doi:10.1038/s41598-017-14750-w)
Supplement: Supplementary file 1 — Mathematical deviations of corneal tangent modulus [file 41598_2017_14750_MOESM1_ESM.pdf]

Title Page

Title: *In vivo* measurement of regional corneal tangent modulus

Author list:

Ying Hon[1], Guo-Zhen Chen[2], Shu-Hao Lu[2], David CC Lam[2], Andrew KC Lam[1]

Laboratory of Experimental Optometry, School of Optometry, The Hong Kong Polytechnic University, Hong Kong

[2]*Department of Mechanical and Aerospace Engineering, The Hong Kong University of Science and Technology, Hong Kong*

Supplementary information

Mathematical deviations of corneal tangent modulus[1]

The basic principle of Goldman applanation tonometry comes from the Imbert-Fick law.[2] Ideally, the IOP is given by

$$\mathrm{IOP} = \frac{F}{A}\,, \tag{1}$$

where *F* is the force applied to the applanation head and *A* is the flattened area of the cornea (Figure 7). The law is valid when the cornea is infinitely thin, perfectly elastic, spherical and dry at the contact surface. Nevertheless, none of the assumptions is true for human corneal tissue. When an indentation probe is in contact with the cornea, the actual balance of force is therefore

$$F + s = A \cdot \mathrm{IOP} + b\,, \tag{2}$$

where *s* is the surface tension of the tear film and *b* is the resistance of the cornea to deformation (Figure 7). The resistance force *b* during indentation of a partial spherical shell by a concentrated force is derived from the Roark's formula,[3] which can be calculated as

$$b = \frac{E \cdot t^2}{a(R - t/2)\sqrt{1 - v^2}}\delta \tag{3}$$

in the absence of surface tension and IOP. The resistance force *b* is dependent on corneal material properties, such as corneal tangent modulus *E*, corneal geometry constant *a*, corneal thickness *t*, corneal radius of curvature *R*, Poisson's ratio of the cornea *v*, and indentation depth *δ*. The value of *a* is determined from *μ*,[3,4]

$$\mu = r_o \left[\frac{12(1 - v^2)}{(r - t/2)^2 t^2}\right]^{1/4} \tag{4}$$

where r_0 is the radius of a circular flat-surface probe that is in full contact with the cornea.

By differentiating equation (2) with respect to indentation depth *δ*, it gives

$$\frac{dF}{d\delta} + \frac{ds}{d\delta} = \frac{d}{d\delta}(A \cdot IOP) + \frac{db}{d\delta}\,. \tag{5}$$

Changes in the surface tension s can be negligible under constant IOP and constant contact area A. After substituting equation (3) into (5), it gives

$$\frac{dF}{d\delta} = \frac{E \cdot t^2}{a(R-t/2)\sqrt{1-v^2}} \,. \tag{6}$$

Rearranging equation (6), the equation for corneal tangent modulus becomes

$$E = \frac{a(R-t/2)\sqrt{1-v^2}}{t^2} \frac{dF}{d\delta} \,. \tag{7}$$

Therefore corneal tangent modulus E at individual-specific IOP can be determined by substituting individual corneal thickness t and curvature R measured using available clinical devices, and individual corneal stiffness $\frac{dF}{d\delta}$ obtained from the CID.

References

1 Ko, M. W., Leung, L. K., Lam, D. C. & Leung, C. K. Characterization of corneal tangent modulus in vivo. *Acta Ophthalmol* **91**, e263-269 (2013).

2 Ethier, C. R. & Simmons, C. A. *Introductory biomechanics: from cells to organisms*. (Cambridge University Press, 2007).

3 Young, W. C. & Budynas, R. G. in *Roark's formulas for stress and strain.* Ch. 13, 610 (McGraw-Hill Book Co., 2002).

4 Reissner E. Stresses and small displacements of shallow spherical shells II. *J Math Phys* **25**, 80-85 (1946).
